# Development of rheumatoid arthritis during the course of gefitinib therapy

**DOI:** 10.1590/S1516-31802009000300013

**Published:** 2009-10-06

**Authors:** Gen Ohara, Hiroaki Satoh, Mika Kohno, Daisuke Goto, Takayuki Sumida, Nobuyuki Hizawa

**Affiliations:** 1 MD. Member of the medical staff, Division of Respiratory Medicine, Institute of Clinical Medicine, University of Tsukuba, Tsukuba-city, Ibaraki, Japan.; 2 MD. Professor, Division of Respiratory Medicine, Mito Medical Center, University of Tsukuba, Mito, Ibaraki, Japan.; 3 MD. Member of the medical staff, Division of Rheumatology Medicine, Institute of Clinical Medicine, University of Tsukuba, Tsukuba-city, Ibaraki, Japan.; 4 MD. Professor, Division of Rheumatology Medicine, Institute of Clinical Medicine, University of Tsukuba, Tsukuba-city, Ibaraki, Japan.; 5 MD. Professor, Division of Respiratory Medicine, Institute of Clinical Medicine, University of Tsukuba, Tsukuba-city, Ibaraki, Japan.

We read with interest the article by del Giglio and Ito on arthralgia as an adverse effect in non-small cell lung cancer (NSCLC) patients treated with gefitinib.^1^ We would like to share our experience.

A 57-year-old woman with no smoking history was admitted because of a nodular lesion that was detected incidentally by a chest radiograph. A chest computed tomography (CT) scan demonstrated a left lung mass with intrapulmonary metastases.

Pathology samples obtained by transbronchial biopsy confirmed adenocarcinoma of the lung. Brain magnetic resonance imaging (MRI) showed three nodules, up to 10 mm in diameter, consistent with metastatic disease. She received gamma-knife radiosurgery. Thereafter, she received 250 mg gefitinib per day. A chest CT scan on day 28 revealed regression of the primary lesion.

Seven months after starting on gefitinib, she showed swelling with pain on movement, accompanied with morning stiffness almost symmetrically in the bilateral metacarpophalangeal and proximal interphalangeal joints. Swelling and pain were observed bilaterally in the knee and ankle joints. These symptoms continued for two months. Laboratory data demonstrated positive inflammatory reactions, including the erythrocyte sedimentation rate (90 mm/1 h) and C-reactive protein (1.70 mg/dl). The rheumatoid factor and matrix metalloproteinase-3 concentrations were 161 U/ml and 74.8 ng/ml, respectively. She was diagnosed as having rheumatoid arthritis, according to the standard classification criteria.^2^

Oral prednisolone was started at an initial dose of 5 mg per day. Sulfasalazine was then started at a dose of 500 mg daily, orally. Soon after this therapy, her arthritis showed improvement and inflammatory reactions decreased. She has since been treated with gefitinib and prednisolone, and has been in a good general condition without recurrence of lung adenocarcinoma and arthralgia due to rheumatoid arthritis for 12 months.

Gefitinib is an oral selective inhibitor of the epidermal growth factor receptor (EGFR) tyrosine kinase, and its use is now being attempted for patients with advanced non-small cell lung cancer (NSCLC).^3^^,^^4^ Our case was a lung adenocarcinoma patient who developed rheumatoid arthritis seven months after starting on gefitinib therapy. In this report, we emphasize two points.

First, prednisolone and sulfasalazine are widely used for treating rheumatoid arthritis, although there are serious possible complications, including infection, gastric ulcer, diabetes, skin eruption, and myelosuppression. The influence of steroids on gefitinib therapy for NSCLC has scarcely been reported.^5^ The initial therapy applied in our case consisted of very low doses of prednisolone and sulfasalazine, because we did not know what influence these drugs might have on gefitinib therapy. We were unable to conclude whether rheumatoid arthritis within a picture of lung cancer treated with gefitinib might response with low-dose prednisolone and sulfasalazine, but this patient’s rheumatoid arthritis showed a good clinical response without increasing the doses of these drugs. Although this clinical experience only consists of a single case, our result suggests that prednisolone and sulfasalazine seem to have neither favorable nor unfavorable effects on gefitinib therapy.

Second, the development of rheumatoid arthritis after starting gefitinib therapy is an extremely rare event and does not depend on previous rheumatic manifestations. Although del Giglio and Ito described mild arthralgia in two NSCLC patients treated with gefitinib,^1^ our assessment is that gefitinib does not work as a “trigger” leading to deregulation of the immune cascade, even in subjects genetically predisposed to develop rheumatoid arthritis.
